# Nivolumab-Induced Warm Autoimmune Hemolytic Anemia and Pancytopenia

**DOI:** 10.7759/cureus.92951

**Published:** 2025-09-22

**Authors:** Matthew Baron, Fnu Amisha, Kiranpreet Kaur, Neha Verma

**Affiliations:** 1 Internal Medicine, Moffitt Cancer Center, Tampa, USA; 2 Oncology, Moffitt Cancer Center, Tampa, USA; 3 Internal Medicine, University of South Florida Morsani College of Medicine, Tampa, USA

**Keywords:** clear cell renal carcinoma, drug-induced pancytopenia, immunotherapy-related adverse events, nivolumab-related adverse events, non-malignant hematology, warm autoimmune hemolytic anemia

## Abstract

Immunotherapy-related adverse events arise from the disinhibition of the body's own immune system. A 76-year-old Caucasian man undergoing treatment with nivolumab monotherapy for metastatic clear cell renal carcinoma presented to our hospital with symptoms of fatigue and easy bruising. Laboratory analysis confirmed warm autoimmune hemolytic anemia with pancytopenia. Bone marrow biopsy demonstrated no evidence of malignancy or marrow failure. He was initiated on guideline-driven therapy with corticosteroids and experienced complete resolution of cytopenias by the three-month follow-up.

## Introduction

Immunotherapy-related adverse events (irAEs) have taken on increasing clinical importance with the expansion of indications for the drug class. The novelty of treatment and breadth of side effects present a diagnostic and treatment challenge for general practitioners whose formal training concluded far before the drug class's introduction. Significant or high-grade adverse events occur in approximately 25% of the treatment population [1]. Herein, we review a rare case of immunotherapy-related pancytopenia with warm autoimmune hemolytic anemia in the setting of nivolumab monotherapy for clear cell renal carcinoma. Current literature estimates the prevalence within the treatment population of immunotherapy-related pancytopenia at less than 1% [1] and immunotherapy-related warm autoimmune hemolytic anemia at approximately 0.25% [2]. The pathophysiology of immunotherapy-related cytopenias involves the inappropriate activation of the body's own immune defenses against either progenitor cells within the bone marrow or mature cells within the peripheral blood. Treatment involves the pharmacological suppression of this overactive immune response, with corticosteroids being the first-line agent. The greatest barrier or challenge to the treatment of the condition is accurate diagnosis, as oncologic patients can have varying degrees of pancytopenia from various causes and the etiology can easily be misdiagnosed if there is insufficient index of suspicion. Demonstration of informative examples within the case literature is essential to highlight both the common and rare examples of irAEs.

## Case presentation

A 76-year-old Caucasian man presented to the emergency room of our tertiary care hospital in 2024 with complaints of dyspnea on exertion, progressive fatigue, and easy bruising of two weeks' duration. His past medical history included metastatic clear cell renal carcinoma, essential hypertension, hypothyroidism, chronic obstructive pulmonary disease, prostate cancer, and benign prostatic hyperplasia. The patient was first diagnosed with renal cell carcinoma in 1999 and underwent a nephrectomy. Later scans revealed metastatic disease in the pancreas, leading to the initiation of sunitinib treatment in 2013. In 2017, treatment was paused, and the patient entered a period of active surveillance. However, the development of pulmonary metastasis in 2021 led to the start of four cycles of combination ipilimumab/nivolumab therapy, followed by continued nivolumab monotherapy thereafter.

The patient denied fever, night sweats, unintentional weight loss, alcohol or any illicit drug use, recent travel, or sick contacts. He denied a prior history of bleeding disorder or bleeding from any other site. On medication review, he denied use of non-steroidal anti-inflammatory drugs (NSAIDS), antiplatelet agents, anticoagulants, or herbal supplements.

On physical examination, there were pallor and areas of ecchymosis to both upper and lower extremities. The remainder of the examination was within normal limits including no evidence of organomegaly or lymphadenopathy. The initial laboratory evaluation is described in Table 1. Complete blood count (CBC) demonstrated pancytopenia with depression of all three major cell lines. Elevated lactate dehydrogenase, indirect bilirubin and reticulocyte count within the high normal range, and depressed haptoglobin level were suggestive of a hemolytic process. Follow-up direct antiglobulin testing was positive for immunoglobulin G (IgG) antibodies consistent with warm autoimmune hemolytic anemia. 

**Table 1:** Hematological parameters at presentation Hematologic results demonstrate pancytopenia with a suspected underlying hemolytic process, which was ultimately confirmed with direct antiglobulin testing. WBCs: white blood cells; MCV: mean corpuscular volume; MCH: mean corpuscular hemoglobin; MCHC: mean corpuscular hemoglobin concentration; AST: aspartate aminotransferase; ALT: alanine aminotransferase; LDH: lactate dehydrogenase; PT: prothrombin time; aPTT: activated partial thromboplastin time

Test	Results	Units	Normal range
WBCs	1.83	k/mcL	4.0-10.9
Absolute neutrophil count	0.26	k/mcL	1.8-7.8
Eosinophils	0.00	k/mcL	0.00-0.45
Basophils	0.00	k/mcL	0.00-0.20
Monocytes	0.26	k/mcL	0.30-0.80
Lymphocytes	1.32	k/mcL	1.10-3.50
Nucleated red blood cells	0.00	k/mcL	0.00-0.10
Hemoglobin	11.0	g/dL	13.4-16.9
MCV	94.5	fL	80.3-94.0
MCH	31.8	pg	27.4-33.4
MCHC	33.6	g/dL	32.0-36.8
Platelet count	50	k/mcL	143-382
Creatinine	0.8	mg/dL	0.7-1.3
Calcium corrected	9.4	mg/dL	8.6-10.2
Alkaline phosphatase	122	U/L	40-130
AST	54	U/L	5-34
ALT	15	U/L	0-55
Total bilirubin	1.3	mg/dL	0.0-1.2
Direct bilirubin	0.6	mg/dL	0.0-0.5
Indirect bilirubin	0.7	mg/dL	≤1.1
LDH	350	U/L	135-225
Haptoglobin	<8	mg/dL	30-200
Corrected reticulocyte count	1.72	%	0.8-1.9
PT	14.5	sec	10.2-12.9
aPTT	30.9	sec	25.1-36.5
Fibrinogen	233	mg/dL	200-393
D-dimer	6574	ng/mL	<500

**Figure 1:**
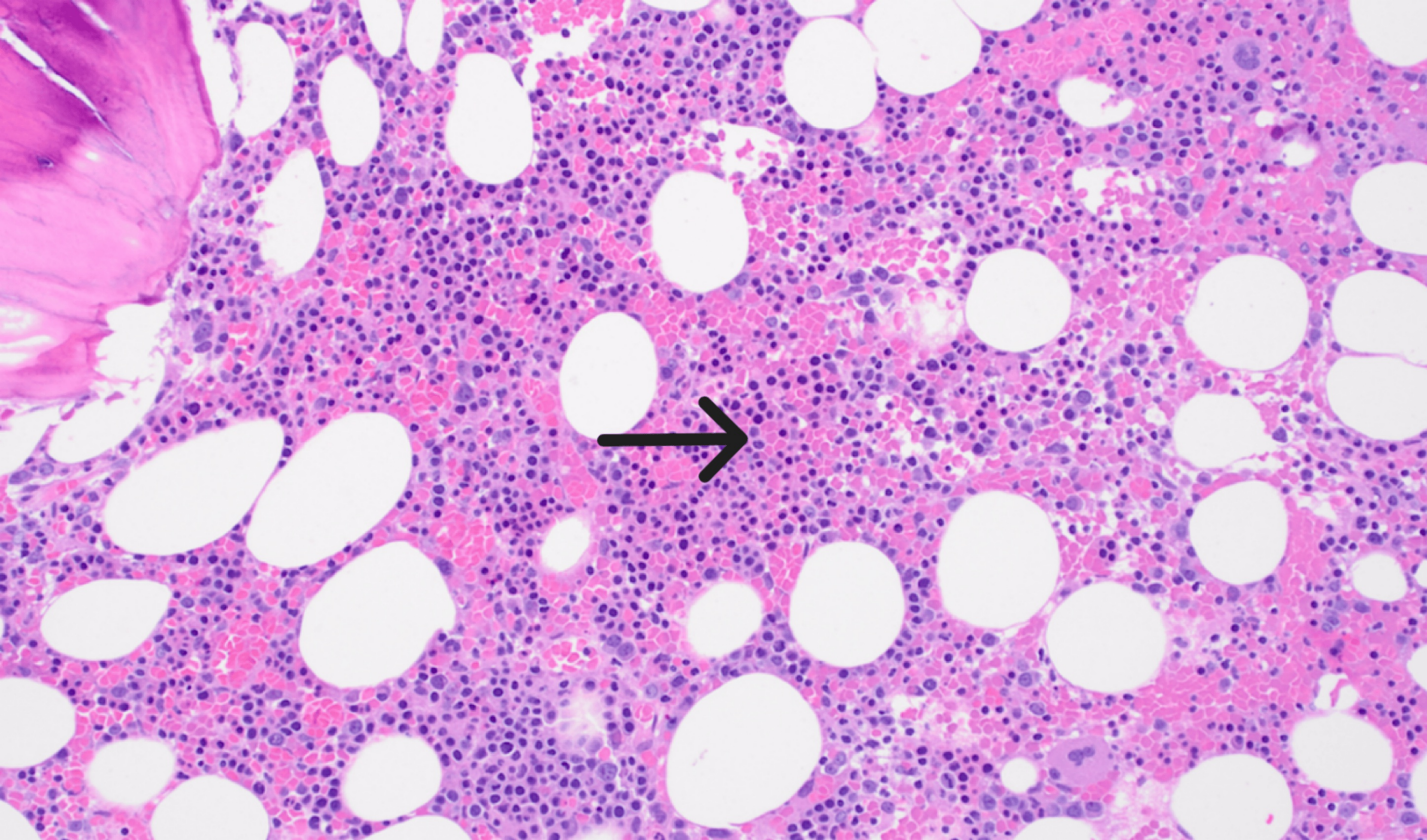
Bone marrow biopsy The arrow demonstrates the healthy bone marrow with an appropriate physiologic increase in trilineage hematopoiesis in response to peripheral pancytopenia.

**Figure 2:**
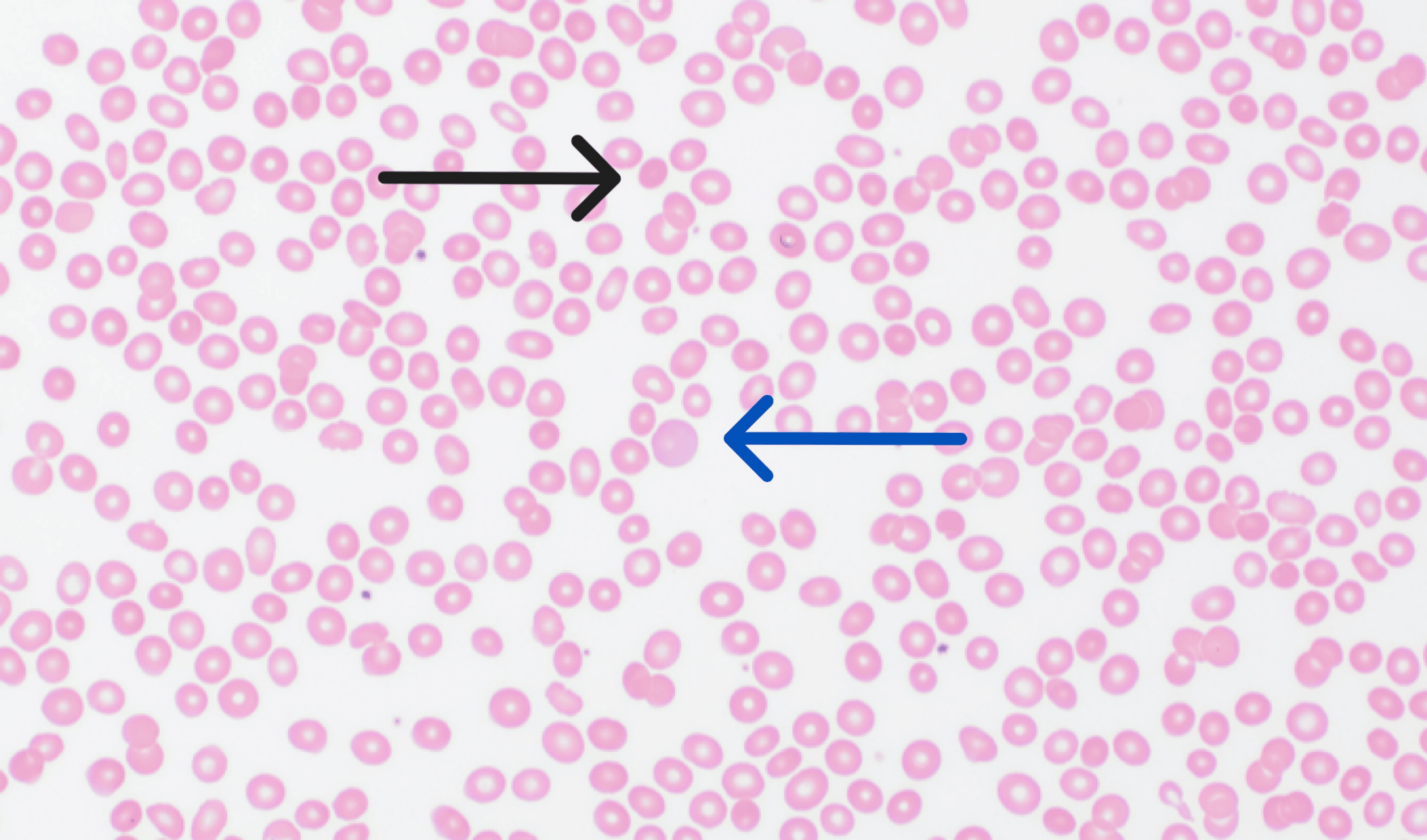
Peripheral smear Peripheral blood smear demonstrates rare spherocytes and mildly increased reticulocytes consistent with warm autoimmune hemolytic anemia. The black arrow highlights a spherocyte resulting from an autoimmune attack of a peripheral red blood cell. The blue arrow demonstrates polychromasia characteristic of immature red blood cells resulting from appropriate reticulocytosis in the setting of anemia.

A retrospective chart review dating back more than two years to the start date of nivolumab monotherapy demonstrated a near-normal CBC with a previous hemoglobin nadir of 13.4 g/dL and a platelet nadir of 136 k/mcL. To further evaluate the cause of pancytopenia, we conducted an additional workup as described in Table 2. Infectious studies did not detect any of the typical viral, bacterial, or fungal etiologies associated with pancytopenia. Nutritional panel showed no evidence of vitamin or mineral deficits and/or excesses typically associated with pancytopenia. Iron studies were consistent with anemia of chronic disease and ruled out iron deficiency. Bone marrow biopsy demonstrated increased trilineage hematopoiesis with reactive features and no overt morphological evidence of an underlying myelodysplastic syndrome, leukemia, or lymphoma (Figure 1). There was no evidence of metastatic involvement from the patient's underlying clear cell renal carcinoma. Peripheral blood smear demonstrated pancytopenia, rare spherocytes, mildly increased reticulocytes, and no blasts (Figure 2). Flow cytometry was unremarkable. 

**Table 2:** Infectious workup and nutritional labs Targeted infectious and nutritional evaluations for pancytopenia demonstrate largely benign results. TIBC: total iron-binding capacity; EBV PCR: Epstein-Barr virus polymerase chain reaction; CMV: cytomegalovirus; HIV: human immunodeficiency virus

Test	Results	Units	Normal range
Iron	37	mcg/dL	65-175
TIBC	87	mcg/dL	250-450
Ferritin	3099	ng/mL	30-400
Vitamin B12	>2000	pg/mL	211-946
Folic acid	17.4	ng/mL	7.0-31.4
EBV PCR	Negative		
CMV PCR	Negative		
Human herpesvirus 6 PCR	Negative		
HIV antibody/antigen	Negative		
Hepatitis B core antibody	Negative		
Hepatitis B surface antigen	Negative		
Hepatitis C antibody	Negative		
1,3-Beta-D-glucan fungitell	Negative		
Histoplasma galactomannan antigen	Negative		
Blood culture	Negative		

**Figure 3:**
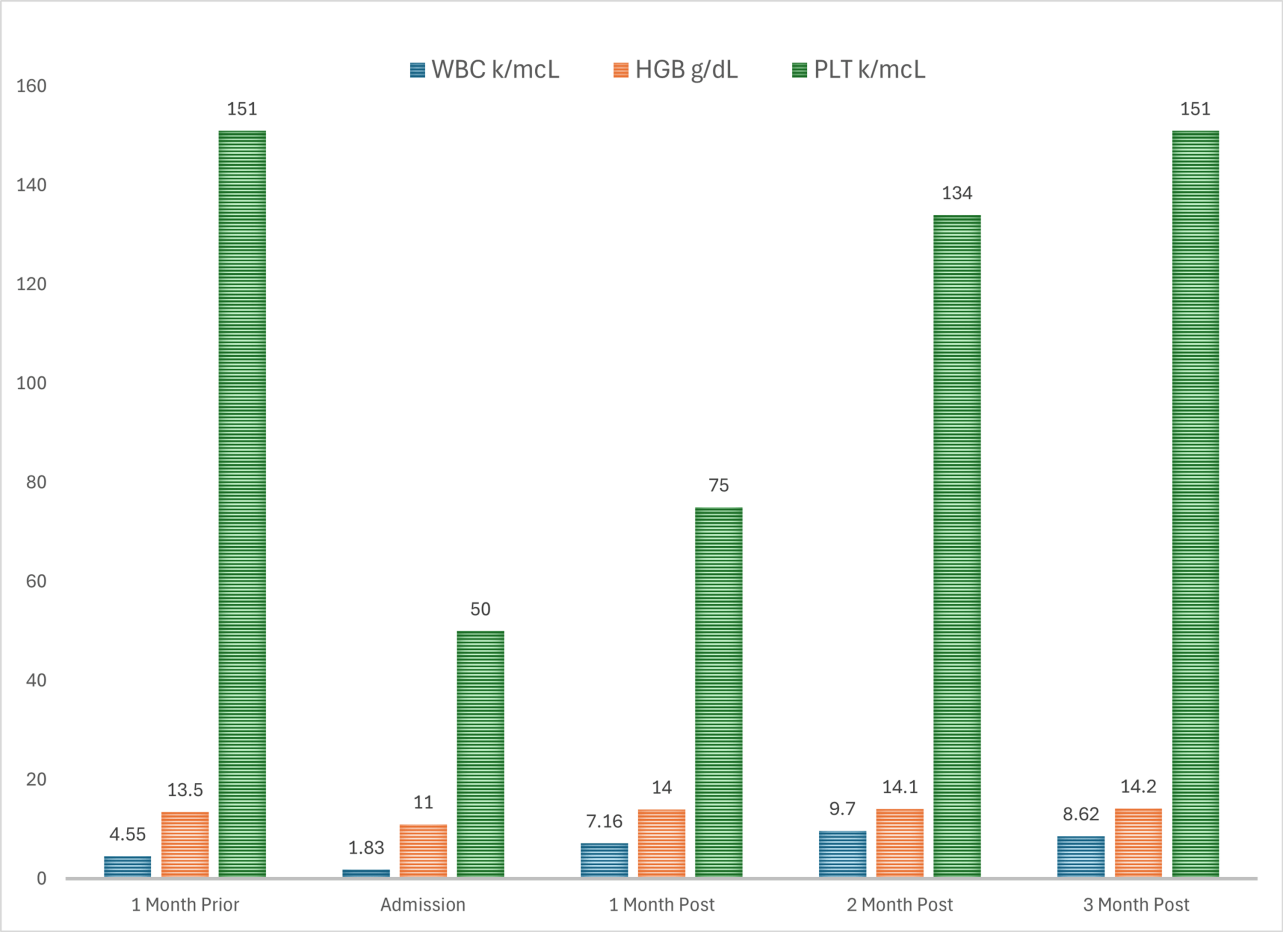
Onset and resolution of pancytopenia The graph demonstrates normal WBC count, Hgb, and Plt count one month prior to admission. Admission labs revealed pancytopenia resulting from nivolumab treatment. Subsequent follow-up studies confirmed count recovery in response to corticosteroid treatment. WBC: white blood cell; Hgb: hemoglobin; Plt: platelet

The patient was initiated on intravenous methylprednisolone 125 mg twice daily for warm autoimmune hemolytic anemia and presumed immunotherapy-related pancytopenia secondary to the immune checkpoint inhibitor nivolumab. He was discharged on prednisone 100 mg daily with a down-titration by 10 mg every week until the prednisone level reached 20 mg, at which time taper was slowed to 5 mg every week until off. Leukopenia and anemia resolved at the one-month follow-up, and thrombocytopenia resolved at the three-month follow-up (Figure 3).

## Discussion

Nivolumab and other programmed cell death protein 1/programmed cell death ligand 1 (PD-1/PD-L1) monoclonal antibodies within the immune checkpoint inhibitor class of pharmaceuticals have an extensive record of irAEs. Literature estimates of the incidence of irAEs vary from study to study, with pooled analysis estimating the overall frequency of high-grade toxicities of any kind at about 25% [1]. irAEs can occur within any organ system of the body, with nivolumab toxicity occurring most commonly in the skin, gastrointestinal tract, and endocrine organs [3].

High-grade hematologic toxicity is rare with isolated cytopenia at around 3.6% and pancytopenia at 0.6% [1]. Immunotherapy-related warm autoimmune hemolytic anemia is rarer still at 0.25% [2]. The time to onset of irAE in this case was also unusual, with the majority of irAEs occurring within the first one to two months of treatment onset, but case reports do demonstrate evidence of onset more than a year after the initiation of treatment or even after treatment termination [3].

Despite its rarity, immunotherapy-related hematologic toxicity must be included in the physician's differentials. Oncologic patients can have multiple causes for cytopenias, ranging from self-resolving etiologies like cytotoxic chemotherapy to intractable etiologies like malignant bone marrow infiltration in the setting of widely metastatic disease. Immunotherapy-related hematologic toxicities are often readily treatable if appropriately identified with resolution rates of up to 60% [4]. 

Patients presenting with pancytopenia carry a poorer prognosis than those with cytopenia limited to a single cell line [4]. In our patient's case, he demonstrated evidence of peripheral blood involvement without significant evidence of central marrow toxicity. It is hypothesized that in patients with hypocellular bone marrow, which is a characteristic of aplastic anemia, activated T cells triggered by nivolumab may have caused damage to hematopoietic stem cells [5]. 

The American Society of Clinical Oncology offers guidelines for immunotherapy interruption and treatment based on the organ system involved and grade of toxicity. Our patient was categorized as grade 3 irAE based on cell counts, and nivolumab was held in accordance with guidelines [6]. Our patient was never reinitiated on immunotherapy, and the literature is scarce regarding the appropriateness of rechallenge after immunotherapy-related hematologic adverse events, but the limited patient samples suggest a recurrence rate for cytopenias of approximately 40% [4].

## Conclusions

Immunotherapy is an ever-expanding modality of treatment in an increasing variety of malignancies. High-grade irAEs will increase in frequency as the treatment population grows. Adverse events can occur anywhere along the treatment timeline, sometimes with significant delay from treatment onset as this case demonstrates. Hematologic toxicity is a rare, but important, subset of irAEs. It can be a diagnostic challenge secondary to the high prevalence of cytopenias in our oncologic treatment population. Clinical discipline is required to appropriately exclude alternative etiologies related to polypharmacy, infection, nutrition, or malignancy. Clinical response rates are strong for those patients promptly diagnosed and appropriately treated with corticosteroids.
